# Salt-Restriction-Spoon Improved the Salt Intake among Residents in China

**DOI:** 10.1371/journal.pone.0078963

**Published:** 2013-11-11

**Authors:** Juan Chen, Ye Tian, Yixing Liao, Shuaishuai Yang, Zhuoting Li, Chao He, Dahong Tu, Xinying Sun

**Affiliations:** 1 Department of Social Medicine and Health Education, School of Public Health, Peking University, Beijing, China; 2 China Center for Health Development Studies, School of Public Health, Peking University, Beijing, China; 3 VIP Medical Service Department, Peking Union Medical College Hospital, Beijing, China; 4 Department of Health Education, Shun Yi Center for Disease Prevention and Control, Beijing, China; 5 Community Medical Center, Beijing Shijitan Hospital, Beijing, China; The Ohio State University, United States of America

## Abstract

**Objective:**

To evaluate the effect of an improved salt-restriction spoon on the attitude of salt-restriction, the using rate of salt-restriction-spoon, the actual salt intake, and 24-hour urinary sodium excretion (24HUNa).

**Design:**

A community intervention study.

**Setting:**

Two villages in Beijing.

**Participants:**

403 local adult residents being responsible for home cooking.

**Intervention:**

Participants were randomly assigned to the intervention group or the control group. Those in the intervention group were provided with an improved salt-restriction-spoon and health education, and were informed of their actual salt intake and 24HUNa. Not any intervention was given to those in the control group.

**Main Outcome Measures:**

The scores on the variables of Health Belief Model, the using rate of salt-restriction-spoon, the actual salt intake, and 24HUNa.

**Analysis:**

Covariance analyses, Chi-square tests, Student’s *t* tests, and repeated measures analyses of variance.

**Results:**

After 6 months of intervention, the intervention group felt significantly less objective barriers, and got access to significantly more cues to action as compared to the control group. The using rate and the correctly using rate of salt-restriction-spoon were significantly higher in the intervention group. The daily salt intake decreased by 1.42 g in the intervention group and by 0.28 g in the control group, and repeated measures analysis of variance showed significant change over time (*F* = 7.044, *P*<0.001) and significant difference between groups by time (*F* = 2.589, *P* = 0.041). The 24HUNa decreased by 34.84 mmol in the intervention group and by 33.65 mmol in the control group, and repeated measures analysis of variance showed significant change over time (*F* = 14.648, *P*<0.001) without significant difference between groups by time (*F* = 0.222, *P* = 0.870).

**Conclusions:**

The intervention effect was acceptable, therefore, the improved salt-restriction-spoon and corresponding health education could be considered as an alternative for salt reduction strategy in China and other countries where salt intake comes mainly from home cooking.

## Introduction

Cardiovascular disease has become one of the leading causes of death worldwide, and hypertension is the major risk factor associated with cardiovascular disease [1], [2], [3], [4], [5]. A survey conducted in 2010 shown that the incidence of hypertension for Chinese people aged over 18 was 33.5% [6]. In China, hypertension accounts for 40% of the deaths and 23% of the health care costs [7].

Meta-analysis has indicated the causal link between salt intake and high blood pressure. A meta-analysis of randomized trial demonstrated that a reduction of salt intake to 6 g/day would lower BP by 7/4 mmHg in hypertensives and 4/2 mmHg in normotensives [8]. In another meta-analysis, a reduction of 100 mmol in daily urinary sodium excretion, that is a reduction of 5.9 g in daily salt intake, would decrease systolic blood pressure by an average of 3.7 mmHg and diastolic blood pressure by an average of 0.9 mmHg in hypertensive subjects [9]. Population-based intervention studies have also shown the effect of salt intake on blood pressure, among which, the most successful one conducted in Portugal achieved a difference of 13/6 mmHg in BP between two groups after two years’ salt-reduction intervention [10].

However, the current situation of salt intake is not satisfactory. According to a survey conducted in Beijing, the estimated average daily salt intake in 2008 was 9.70 for urban residents and 13.28 for rural residents [11]. A recent study published in the Journal of the American Dietetic Association stated that the mean sodium intake of Chinese was 3991 mg/person/day (equivalent to mean salt intake of 10.15 g/person/day), and the mean northern sodium intake was 4733 mg/person/day, notably higher than southern 2491 mg/person/day (equivalent to mean salt intake of 12.04 g/person/day and 6.34 g/person/day, respectively) [12]. A study conducted in Beijing shown the median salt intake of residents aged over 6 to be 12.1 g, higher than the national level [13]. Another survey conducted in Beijing stated that the salt intake was 165.79 mmol for urban residents and 226.80 for rural residents [11].

In contrast with western countries, most (76%) dietary sodium was from the salt added in home cooking [12], so a public campaign is needed to encourage consumers to use less salt [8]. The Chinese government has taken some measures to reduce the average salt intake from home cooking, among which, salt-restriction spoon is a commonly adopted one. With a 5 cm handle, a 1.1 cm caliber, and a volume to hold 2 g salt, the salt-restriction-spoon was designed to help people calculate the amount of salt they used. For example, a family with four members should eat no more than 24 g salt per day, equal to 12 spoons of salt. So the housewife should use 4 spoons of salt per meal.

Unfortunately, many people who have received the salt-restriction spoons do not use them or cannot use them correctly. A survey conducted in Beijing indicated that the owning rate, using rate and correctly using rate of salt-restriction spoon were 61.8%, 47.7% and 17.1% respectively, and that 69.7% of those who did not use a salt-restriction spoon considered the current spoons to be inconvenient for use [14]. The previous survey conducted by our research group shown that the receiving rate and using rate of salt-restriction spoon were 72.1% and 32% respectively, and 77.5% of the respondents did not know the right usage of salt-restriction spoon [15]. According to these two surveys, the first three reasons for not using a salt-restriction spoon were: (1) the spoon was not convenient for use, (2) citizens did not realize the importance of salt reduction and (3) citizens were not willing to abandon the habit of eating salty food [14], [15]. The features that made the salt-restriction spoon so unwelcome included: (1) the diameter of the spoon was too small, (2) the handle of the spoon was too short, and (3) the unbent handle made it not pleasing to the eyes [14], [15], [16]. According to our previous analysis, objective barrier of salt-reduction, such as the design defects of a spoon, was the most important determinants of salt-restriction-spoon using behavior [17].

Since that people’s unwillingness to use the salt-restriction spoon can be largely attribute to the design defects that make it inconvenient for use, and that rural residents’ salt intake situation is more worrying as compared to urban residents, this study was designed to improve the current salt-restriction-spoon, and to evaluate the effect of the improved salt-restriction spoon on the attitude of salt-restriction, the using rate of salt-restriction-spoon, the actual amount of salt intake, and 24-hour urinary sodium excretion (24HUNa) of rural residents.

## Materials and Methods

### Ethics Statement

Written informed consent from all the participants was obtained at enrolment, and the study protocol was approved by the Ethical Committee on Biomedical Research Involving Human Subjects of the Health Science Center, Peking University.

### Study Design

Conducted during JUN 2012 and JAN 2013 in the suburban area of Beijing, this community intervention study lasted for 7 months, with 1 month to collect the baseline information and 6 month to evaluate the effect of intervention. Eligible participants were randomized into the interventional group or the control group. Allocation was blinded to the participants during the entire study period. All the baseline information was collected by JUL 2012, after when the intervention measures were given to the participants in the intervention group, while no intervention was given to those in the control group. Four follow-up visits were arranged for all the participants, with the final visit in JAN 2013. The detailed schedule was presented in Table 1.

**Table 1:** Time schedule.

	2012					2013
	JUN	JUL	AUG	SEP	NOV	JAN
	Visit −1	Visit 0	Visit 1	Visit 2	Visit 3	Visit 4
Enrolment	√					
Questionnaire	√					√
Weight salt	√	√	√	√	√	√
Collect urine		√		√	√	√
Hand out spoon^*^		√				
Health education^*^		√	√	√	√	√

Note: ^*^only for the intervention group.

### Sample Selection

A stratified cluster random sampling method was applied to enroll 403 rural residents in two villages in Beijing. To be eligible for the study, participants should meet all the following criteria: (1) being a usual resident in one of the two villages; (2) aged over 18; (3) being responsible for home cooking; (4) without any severe complications of hypertension or diabetes; (5) with clear consciousness; (6) usually ate at home.

### Intervention Measures

After baseline information had been collected, the improved salt-restriction spoons were handed out to the participants in the intervention group. With the volume to hold 2 g salt, the spoon improved by us has the following features as compared to the original one sent to the Beijing citizens in 2007–2008: a longer and curved handle (9.5 cm), a wider caliber (2.2 cm), a 1 g scale on the internal surface of the spoon, a matched salt shaker with scales on one side surface, exchange table for calculating the amount of salt from other sources (such as soy sauce) on the other side surface, and salt-reduction slogan on the top surface. The improved salt-restriction spoon has gained a patent in China.

Besides the improved spoon, some simple health education was given to the intervention group at each follow-up visit, which was focus on the correct usage of the spoon and the method of calculating the amount of salt one should eat.

In addition, the participants in the intervention group were informed promptly of their actual salt intake and 24-hour urinary sodium excretion obtained at each visit. The participants in the control group could also know their 24-hour urinary sodium if they asked about it.

### Measurements

Altogether four endpoints were obtained to evaluate the effect of intervention: change in the scores of variables in the HBM and change in salt-restriction-spoon using behavior measured by a questionnaire, change in salt intake obtained by salt weighting, and change in 24HUNa obtained by 24-hour urine collection.

#### Questionnaire

As depicted in a paper being submitted [17], a self-designed questionnaire was structured based on the HBM, in which respondents’ salt-restriction-spoon using behavior (the frequency and method of using a salt-restriction spoon), demographic characteristics (age, degree of education, family income, living places), perceived susceptibility to hypertension (α = 0.894), perceived severity of hypertension (α = 0.807), perceived benefits of salt-restriction (α = 0.803), perceived objective barriers of salt-restriction (the barriers related to the design defect and the usage of the salt-restriction-spoon, α = 0.838), perceived subjective barriers of salt-restriction (the barriers related to the negative feeling of salt-intake reduction, α = 0.680), self-efficacy (α = 0.912, knowledge of hypertension (α = 0.766) and cues to action (α = 0.642) were tested. This instrument has good reliability (Cronbach α = 0.757) and validity (accumulative contribution rate = 63.50%). The questionnaire survey was conducted in the participant’s home at baseline and the final visit (see Table 1).

#### Salt intake tracking

At baseline and each follow-up visit, the salt that had been unpackaged was weighted (see Table 1), and a dietary recording sheet was sent to each participant. The participants were told to record the number of people eating at home and the weight of salt that was opened from the current visit to the next visit on the dietary recording sheet. Taking the baseline data as an example to illustrate the calculation of average daily salt intake, the baseline average daily salt intake of a participant (S_JUN-JUL_) was the total amount of salt eating by a family (weight of salt in JUN [W_JUN_]+weight of salt opened during JUN and JUL [△W_JUN-JUL_] – weight of salt in JUL [W_JUL_]) divided by the number of days in this period (D_JUN-JUL_), and then divided by the average number of people eating at home per day (N). That is: S_JUN-JUL_ = [(W_JUL_+ △W_JUN-JUL_ – W_JUN_)/D_JUN-JUL_]/N. The average daily salt intake during other 4 periods (S_JUL-AUG_, S_AUG-SEP_, S_SEP-NOV_, S_NOV-JAN_) was calculated in the same way.

#### Urine collection and determination of sodium excretion

The urine samples were collected for 4 times, with those collected in JUN 2012 to be the baseline samples (see Table 1). The 24-hour urine was collected into plastic containers from 07∶00 to 07∶00 the next morning, under normal living conditions. The percentage of collected 24-hour urine was ascertained from the participants when they returned the containers and the actual urinary volume was measured, which can be used to calculate the total urinary volume. An AC9000 electrolytic analyzer produced by Jiangsu Audicom Medical Technology and the iron selective electrode method were used to determine urinary sodium concentrations. The 24HUNa was calculated with the formula: 24HUNa = Sodium concentration × total urinary volume.

### Statistical Analysis

Data was processed by EpiData 3.0 and analyzed by SPSS 17.0. Scale and factor analyses were conducted to verify the reliability and validity of the questionnaire. Descriptive analyses, *t* tests and *Chi-square* tests were implemented to examine the demographic features. Using baseline scores of the variables in the HBM as covariants, covariance analyses were conducted to examine the change in the scores of the variables in the HBM. Chi-square tests were used to examine the change of salt-restriction-spoon using behavior. Student’s *t* tests, covariance analyses and repeated measures analyses of variance were implemented to explore the change of salt intake and 24HUNa. In examining the scores of variables in the HBM, the participants who finished both the baseline and the final questionnaire survey were included. In examining the salt intake, the participants whose salt was weighted at all the six visits were included. In examining 24HUNa, the participants whose urine samples were collected at four visits were included. All p-values were two-sided and the p<0.05 level was set as statistical significance.

## Results

### Participant Flow and Follow Up

**Figure 1:**
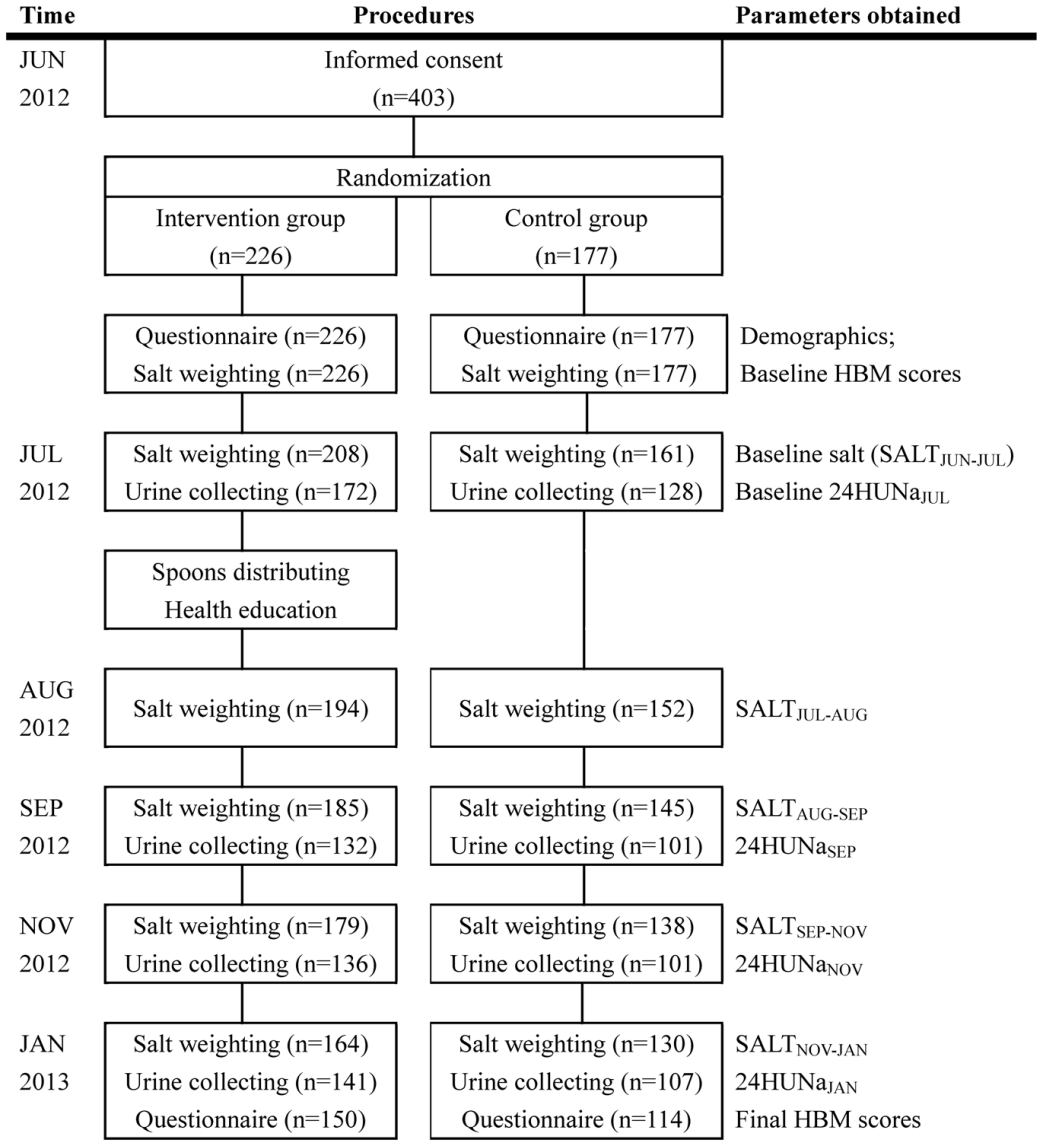
Study flow diagram.

Informed consents were obtained in JUN 2012 from 403 participants, who were randomly assigned to the intervention group and the control group (Figure 1). All of the 403 participants finished the baseline questionnaire. The participants were entitled with the right to withdraw from the study whenever and for whatever reason. “Salt weighting” and “urine collecting” were not compulsory, so data on salt and 24HUNa were not collected for all the participants, and the number of people participated in “salt weighting” and “urine collecting” differed at each visit. At each follow-up visit, only those who had their salt weighted can have their urine collected and finish the questionnaire (at final survey), therefore, the number of participants in corresponding groups who had their salt weighted was more than those who had their urine collected and those who finished the final questionnaire survey (Figure 1). No death happened to any of our participants during the follow-up period. The reasons for withdrawing from the study included: 1) participants’ unwillingness to continue; 2) moving to the urban areas where the heating facilities were more advanced in winter.

Among all the participants, 150 in the intervention group and 114 in the control group finished both the baseline and the final questionnaire survey; 164 in the intervention group and 130 in the control group had their salt weighted at all the 6 visits; 99 participants in the intervention group and 74 in the control group had their urine sample collected at 4 visits.

### Characteristics

According to Table 2, both baseline and final survey noted that the participants in the intervention group had older age and higher family income, and no significant difference in gender, education background, and the prevalence of hypertension exist between groups, which means that the characteristics of those who withdrawn from the study were similar in the intervention group and the control group. Most participants were female and have an educational level of junior middle school graduate.

**Table 2:** Characteristics of participants.

	Baseline				Final			
	Intervention	Control			Intervention	Control		
	(n = 226)	(n = 177)	t or χ^2^	*P value*	(n = 150)	(n = 114)	t or χ^2^	*P value*
Age	54.69±12.30	51.90±13.54	2.151	0.032	57.05±10.58	53.32±11.70	2.710	0.007
Gender			2.712	0.100			1.735	0.188
Male	56 (24.8)	57 (32.2)			34 (22.7)	34 (29.8)		
Female	170 (75.2)	120 (67.8)			116 (77.3)	80 (70.2)		
Education			1.638	0.651			1.649	0.648
< Junior middle school graduate	59 (26.1)	40 (22.6)			41 (27.3)	32 (28.1)		
Junior middle school graduate	125 (55.3)	96 (54.2)			84 (56.0)	59 (51.8)		
Senior middle school graduate	34 (15.0)	32 (18.1)			23 (15.3)	19 (16.7)		
>Senior middle school graduate	8 (3.5)	9 (5.1)			2 (1.3)	4 (3.5)		
Income			8.730	0.033			11.150	0.011
∼999	63 (27.9)	33 (18.6)			50 (33.3)	18 (15.8)		
∼2999	84 (37.2)	58 (32.8)			48 (32.0)	41 (36.0)		
∼4999	58 (25.7)	61 (34.5)			36 (24.0)	40 (35.1)		
5000+	21 (9.3)	25 (14.1)			16 (10.7)	15 (13.2)		
Hypertension history			1.875	0.171			0.868	0.351
Yes	81 (35.8)	52 (29.4)			57 (38.0)	37 (32.5)		
No	145 (64.2)	125 (70.6)			93 (62.0)	77 (67.5)		

### Intervention Effect

#### Change in the scores of variables in the HBM

Table 3 shows the change in the scores of variables in the HBM. As compared to the control group, the intervention group felt significantly less objective barriers of salt-reduction, and got access to significantly more cues to action after intervention. No significant difference in the change of scores of other variables in the HBM was found between the two groups.

**Table 3:** Change in the scores of variables in the HBM.

		Intervention	Control		
Items	Time	(n = 150)	(n = 114)	*F*	*P*
Susceptibility	Corrected baseline	13.96	13.96	3.393	0.067
	Final	14.88±0.36	13.87±0.41		
Severity	Corrected baseline	23.98	23.98	0.040	0.841
	Final	21.71±0.40	21.83±0.46		
Benefits	Baseline	25.11±3.51	25.19±3.31	0.385	0.535
	Final	25.45±3.57	24.70±3.83	2.650	0.105
Subjective barriers	Corrected baseline	13.06	13.06	3.176	0.076
	Final	12.71±0.33	13.60±0.37		
Objective barriers	Corrected baseline	27.54	27.54	221.725	0.000
	Final	21.95±0.57	25.97±0.65		
Self-efficacy	Baseline	37.63±5.47	37.50±5.64	0.010	0.920
	Final	38.19±5.99	37.59±5.72	0.673	0.413
Knowledge	Corrected baseline	3.13	3.13	0.653	0.420
	Final	2.98±0.12	2.84±0.13		
Cues to action	Corrected baseline	2.80	2.80	7.143	0.008
	Final	3.58±0.12	3.11±0.13		

Note: Analysis of covariance was conducted to evaluation the effect of intervention on the scores of susceptibility, severity, subjective barriers, objective barriers, knowledge, and cues to action, the final scores of which were expressed as means±standard error, and the baseline scores of which were expressed as the corrected value. Because the data of benefits and self-efficacy did not meet the requirements for analysis of covariance, analysis of variance was conducted to evaluation the effect of intervention on the two variables, the baseline and final score of which were expressed as means±standard deviation.

#### Change in salt-restriction-spoon using behavior

Table 4 shows the change in salt-restriction-spoon using behavior. Before intervention, the owning rate, using frequency and the correctly using percentage of salt-restriction-spoon were similar between the two groups. After intervention, the owning rate, using frequency and the correctly using percentage of salt-restriction-spoon were significantly higher in the intervention group than in the control group. Before intervention, 26.1% of the participants in the intervention group often or daily use a salt-restriction-spoon, and 13.3% can use it correctly. After intervention, 67.3% of the participants in the intervention group often or daily use a salt-restriction-spoon, and 37.3% can use it correctly.

**Table 4:** Change in the owning rate, using frequency and using method of salt-restriction-spoon.

	Baseline				Final			
	Intervention	Control	?^2^	*P value*	Intervention	Control	?^2^	*P value*
	(n = 226)	(n = 177)			(n = 150)	(n = 114)		
Salt-restriction-spoon			0.001	0.971			43.860	0.000
Yes	126 (55.8)	99 (55.9)			130 (86.7)	56 (49.1)		
No	100 (44.2)	78 (44.1)			20 (13.3)	58 (50.9)		
Using frequency			4.106	0.392			40.207	0.000
Never	138 (61.1)	102 (57.6)			26 (17.3)	57 (50.0)		
Seldom	11 (4.9)	15 (8.5)			9 (6.0)	12 (10.5)		
Sometimes	18 (8.0)	13 (7.3)			14 (9.3)	8 (7.0)		
Often	12 (5.3)	15 (8.5)			35 (23.3)	9 (7.9)		
Everyday	47 (20.8)	32 (18.1)			66 (44.0)	28 (24.6)		
Using method			1.406	0.495			10.121	0.001
Right	30 (13.3)	20 (11.3)			56 (37.3)	22 (19.3)		
Wrong	196 (86.7)	157 (88.7)			94 (62.7)	92 (80.7)		

#### Change in salt intake

**Figure 2:**
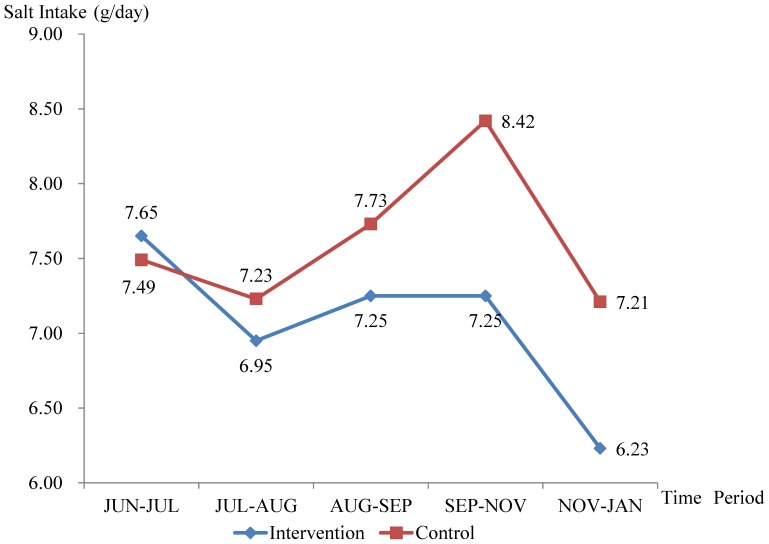
Between-group comparison of the salt intake in five periods of time.

As shown by Table 5, the daily salt intake decreased by 1.42 g in the intervention group and by 0.28 g in the control group after 6 months. Figure 2 shows that the salt intake in both groups declined in the first month of intervention, climbed in the second to fourth months of intervention, and declined again in the last two months of intervention. Repeated measures analysis of variance showed significant change over time (*F* = 7.044, *P*<0.001), and significant difference between groups by time (*F* = 2.589, *P* = 0.041), indicating that the salt intake declined more for the intervention group than for the control group. Student’s t tests showed cross sectional difference between the two groups only in the last two periods of time. Analyses of covariance were conducted to compare the difference between groups, in which group was set as the independent variable, SALT_JUL-AUG_, SALT_AUG-SEP_, SALT_SEP-NOV_ and SALT_NOV-JAN_ were set as the dependent variables respectively, and SALT_JUN-JUL_ was set as a covariate. Consistent with the results of t test, analyses of covariance shown between-group difference only in SALT_SEP-NOV_ (*F* = 7.278, *P* = 0.007) and SALT_NOV-JAN_ (*F* = 7.457, *P* = 0.007). These results indicate that the effect of intervention became more and more obvious with the development of our project.

**Table 5:** Change in the salt intake and 24HUNa.

		JUN-JUL	JUL	JUL-AUG	AUG-SEP	SEP	SEP-NOV	NOV	NOV-JAN	JAN
Saltintake^a^ (g)	Intervention(n = 164)	7.65±4.34		6.95±4.05	7.25±3.89		7.25±3.49		6.23±3.66	
	Control(n = 130)	7.49±4.86		7.23±4.07	7.73±4.75		8.42±5.28		7.21±4.17	
	*t*	0.290		−0.593	−0.956		−2.175		−2.144	
	*P*	0.772		0.554	0.340		0.031		0.033	
24HUNa^b^(mmol)	Intervention(n = 99)		204.28±105.95			206.10±100.54		168.71±75.73		169.44±92.91
	Control(n = 74)		231.34±96.70			240.89±101.58		189.78±77.77		197.69±100.26
	*t*		−1.725			−2.242		−1.789		−1.913
	*P*		0.086			0.026		0.075		0.057

Note: ^a^Please refer to ***“Salt intake tracking”*** for the calculation method of salt intake. The data on salt intake refers to the average daily salt intake in a period of time, not including the condiments that are high in salt, such as soy sauce, monosodium glutamate, salted vegetable, etc.

b Please refer to ***“Urine collection and determination of sodium excretion”*** for the calculation method of 24HUNa. The data on 24HUNa refers to the 24HUNa on the day when the urine was collected, not the average 24HUNa in a period of time.

#### Change in 24HUNa

**Figure 3:**
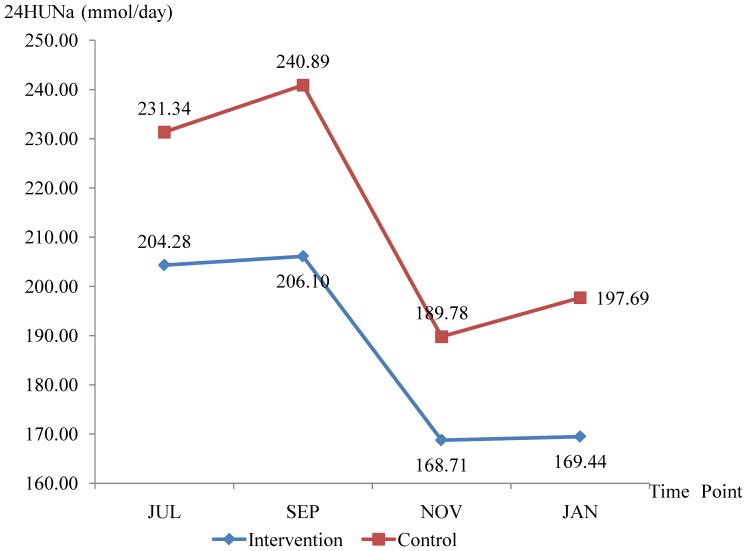
Between-group comparison of the 24HUNa at four time points.

24HUNa was negatively correlated with age (Pearson correlation = −0.215 for 24HUNa at baseline survey, *P*<0.01) and the baseline 24HUNa was higher for the control group than the intervention group (Figure 3 and Table 5). As shown in Table 5, 24HUNa decreased by 34.84 mmol (equal to a salt reduction of 2.04 g) in the intervention group and by 33.65 mmol (equal to a salt reduction of 1.97 g) in the control group after 6 months. Figure 3 shows that during the intervention period the 24HUNa increased slightly in the first two months, declined steeply in the third and fourth months, and increased slightly again in the last two months. Repeated measures analysis of variance showed significant change over time (*F* = 14.648, *P*<0.001), but no significant difference between groups by time (*F* = 0.222, *P* = 0.870), indicating that the level of 24HUNa changed with time similarly in the two groups. Student’s *t* tests showed a significantly cross sectional difference between groups at the second time point only, namely 24HUNa_SEP_. Analyses of covariance were conducted to compare the difference between groups, in which group was set as the independent variable, 24HUNa_SEP_, 24HUNa_NOV_ and 24HUNa_JAN_ were set as the dependent variables respectively, and 24HUNa_JUL_ was set as a covariate. Although significant reduction in 24HUNa was observed in the intervention group, no between-group difference was observed.

## Discussion

This is an attempt of health promotion in suburban areas of Beijing to achieve a population-wide reduction in salt intake, the intervention effect of which was comprehensively evaluated at the levels of perception, behavior and physiology. Neither salt tracking nor 24-hour urine collecting is an easy work, especially for such a long time. In order to make the data as accurate as possible, we tried to help the participants to comply with our work by giving them a user-friendly record, reminding them to record the right information, and calling them before the day of urine collection in case that they forget to collect 24-hour urine. Previous study in 2008 has shown that the 24UHNa for people in urban areas, suburban areas and rural areas of Beijing were 165.79 mmol, 200.43 mmol and 226.80 mmol [11]. Our survey site was located in the suburban area of Beijing, and the baseline 24HUNa for our participants was near to that in the previous study, indicating that the data collected in this study was reliable. The baseline daily salt intake was 7.65 g for the intervention group and 7.49 g for the control group, equal to a sodium intake of 130.77 mmol and 128.03 mmol. The reason that the amount of sodium intake was less than the amount of sodium excretion is because that only salt, the main source of sodium, was weighted in our study, but there are still other sources of sodium such as soy sauce, sodium bicarbonate, salted eggs, monosodium glutamate, and so on [12].

At the level of perception, it’s not surprising that only the scores of objective barriers and cues to action were significantly increased for the intervention group as compared to the control group, since that our intervention measures focused on reducing the objective barriers of salt reduction by giving the participants in the intervention group an improved salt-restriction-spoon and teaching them how to use it. Previous studies have found barriers to be one of the most important predictors of health behaviors, so reducing barriers of salt-reduction would bring us closer to the success in promoting people’s health behavior [18].

Consistent with the reduced objective barrier and increased cues to action, more participants in the intervention group used the improved salt-restriction-spoon frequently and correctly, indicating that the intervention measures were effective in changing one’s behavior. It could be considered to popularize the improved salt-restriction-spoon in other places, as well as other countries where the salt intake of people comes mainly from home cooking, such as those located in Africa [19]. It should be noted that nearly half of those who used a salt-restriction-spoon frequently used it correctly after intervention, which means that our health education was far from enough.

The salt intake mentioned in this paper refers to the salt added in home cooking, not including that from other sources, such as pickles, soy sauce, monosodium glutamate etc. The increase in salt intake from summer to autumn and the decrease in salt intake from autumn to winter may be attributed to the seasonal factors. In summer, Chinese people like to eat porridge with pickles. In autumn, large amount of salt may be used to preserve vegetables for people to eat in winter, which is especially true for rural areas in Northern China, because it’s inconvenient for people there to grow or bur fresh vegetables in winter. Based on the above-mentioned factors, it’s reasonable that the salt used in summer and winter was less than that in autumn. Ruling out the influence of the seasonal factors, the more obvious salt reduction for the intervention group supported the effect of the intervention measures.

As to the results of 24HUNa, the most probable reason for the baseline 24HUNa in the control group being quite high compared to the intervention group would be that participants in the control group were relatively younger than those in the intervention group. As mentioned in the result, 24HUNa was negatively correlated with age. This may be attributed to the better health awareness of the older people who have received large amount of health education and experienced the pain of illnesses. The lower baseline salt intake for the control group in Figure 2 does not mean that participants in the control group took less sodium, because only the kitchen salt added in home cooking was weighted. Younger people may eat more condiment as compared to the older ones, therefore, it is not surprising that the baseline 24HUNa was obviously higher but the baseline salt intake was slightly lower for the control group than for the intervention group.

Although no significant difference was observed in the change of 24HUNa between the two groups, it does not mean that our intervention measures were ineffective. Because the participants in the intervention group and the control group lived in the same village, there would be information contamination between the two groups. In addition, even if no intervention measures were given to the control group, the fact that our investigators went to their home to weight salt and collect urine every one or two months would make them more conscious of the role of salt in one’s health, and thus inevitably remind them to seek for more information on salt, and consequently change their salt-reduction behavior. In addition, some participants in the control group who asked about the laboratory report were also informed of their 24HUNa, which is another factor that may reduce their salt intake. Another survey in which the net intervention effect was a non-significant change in sodium excretion also mentioned these possibilities [20].

It should be noted that the results of salt intake and 24HUNa over the duration of study were discrepant. The measured salt intake increased but the 24HUNa decreased from September to November, and similarly, the measured salt intake decreased but the 24HUNa didn’t from November to January. As mentioned above, this may probably because that some salt was used to preserve vegetables in autumn, and the preserved vegetables were eaten in winter. Since that a large part of the preserved vegetables had not been eaten in autumn, the amount of salt that had been eaten during autumn was actually less than that had been used during this period of time. In winter, less fresh and more preserved vegetables would consumed, leading to a reduction of salt added in home cooking, but not a reduction in 24HUNa. In consideration of these factors, the results of 24HUNa would be more reliable. Assuming that similar proportions of salt were used to preserve vegetables between the two groups, the more obvious reduction in the intervention group compared to the control group at least indicated that the salt added in home cooking was reduced as compared to the control group.

The results that the participants in the intervention group used significantly less salt but did not excrete significantly less urine salt suggested that the improved salt-restriction-spoon did reduce the salt added in home cooking, but did not reduce the salt from other sources. Although an exchange table for calculating the amount of salt in other sources was printed on the side surface of the salt shaker, the print problem make it difficult for the residents to read these information, In addition, we did not emphasized enough on reducing the salt from other sources. Before the improved salt-reduction-spoon was promoted in larger geographic areas, it should be made to be with higher quality, and with a corresponding instruction for use.

Changing one dietary habit is not an easy work [20]. Salt-reduction-spoon is just a tool for helping people calculating the amount of salt and reminding them the importance of salt reduction. Based on the results of this study, the improvements on the spoon did enhance the using rate and correctly using rate, and reduce people’s salt intake, but it’s far from enough to just rely on this simple tool. Even if the residents added less salt in home cooking, they will eat more salt in other ways if their taste keeps unchanged. Therefore, other combined approach, such as low-sodium salt and health education, should be carried out simultaneously with the improved salt-restriction-spoon. What’s more, the change in taste needs long-term intervention.

## Strengths and Limitations

This is the first attempt of tracking the salt consumption of so many people for such a long time, including the first months to collect the baseline salt intake and the later six months to collect the salt intake during the intervention period. In addition, the 24HUNa was collected at four time points. This program was complex and beset by many practical difficulties, but efforts were made to overcome these difficulties and important findings were produced. The evaluation at four levels, namely the awareness of salt reduction, the salt-reduction behavior, the actual salt intake and the 24HUNa, could reflect the interventional effect more comprehensively.

There were several limitations to our study. First, the participants in the intervention group and the control group lived in the same place, causing information contamination to this study, which is probably a reason for the reduction of 24HUNa in the control group. For the sake of ethics requirements, the participants in the control group could be informed of the result of 24HUNa if they took the initiative to ask about it, which may be another reason for the reduction of 24HUNa in the control group. The above-mentioned two points may underestimate the intervention effect. Second, the salt added in home cooking was used to represent the salt intake of people, leaving the salt from other sources uncalculated, such as soy sauce, monosodium glutamate. People’s eating habits in different seasons were not comprehensively considered before conducting the survey, making the evaluation of salt intake not accurate enough. Third, the intervention period was not long enough to change people’s taste, so longer intervention should be carried out in practical context.

## Conclusions

In conclusion, the feasibility of using improved salt-restriction-spoons in general population was found to be good, and their use for six months produced a significant reduction in the objective barriers of salt-restriction, a significant increase in the cues to actions of salt-restriction, a significantly higher using rate and correctly using rate of salt-restriction-spoon, a daily salt reduction of 1.42 g, and a 24HUNa reduction of 34.84 mmol. Therefore, the improved salt-restriction-spoon could be considered as an alternative for salt reduction strategy in China and other countries where salt intake comes mainly from home cooking.
